# On the nonlinear dynamics of a piezoresistive based mass switch based on catastrophic bifurcation

**DOI:** 10.1007/s10999-023-09650-z

**Published:** 2023-02-14

**Authors:** Saber Azizi, Hadi Madinei, Hamed Haddad Khodaparast, Shirko Faroughi, Michael I. Friswell

**Affiliations:** 1 Urmia University of Technology, Urmia, Iran; 2 Swansea University, Swansea, UK

**Keywords:** Mass sensor, Nonlinearity, Piezo resistivity, Bifurcation, Sensitivity, Cantilever

## Abstract

This research investigates the feasibility of mass sensing in piezoresistive MEMS devices based on catastrophic bifurcation and sensitivity enhancement due to the orientation adjustment of the device with respect to the crystallographic orientation of the silicon wafer. The model studied is a cantilever microbeam at the end of which an electrostatically actuated tip mass is attached. The piezoresistive layers are bonded to the vicinity of the clamped end of the cantilever and the device is set to operate in the resonance regime by means of harmonic electrostatic excitation. The nonlinearities due to curvature, shortening and electrostatic excitation have been considered in the modelling process. It is shown that once the mass is deposited on the tip mass, the system undergoes a cyclic fold bifurcation in the frequency domain, which yields a sudden jump in the output voltage of the piezoresistive layers; this bifurcation is attributed to the nonlinearities governing the dynamics of the response. The partial differential equations of the motion are derived and discretized to give a finite degree of freedom model based on the Galerkin method, and the limit cycles are captured in the frequency domain by using the shooting method. The effect of the orientation of the device with respect to the crystallographic coordinates of the silicon and the effect of the orientation of the piezoresistive layers with respect to the microbeam length on the sensitivity of the device is also investigated. Thanks to the nonlinearity and the orientation adjustment of the device and piezoresistive layers, a twofold sensitivity enhancement due to the added mass was achieved. This achievement is due to the combined amplification of the sensitivity in the vicinity of the bifurcation point, which is attributed to the nonlinearity and maximizing the sensitivity by orientation adjustment of the anisotropic piezoresistive coefficients.

## Introduction

The last decade has witnessed an ever-increasing demand for the application of microelectromechanical systems (Bhattacharyya et al. 2008; Azizi et al. 2022; Azizi et al. 2014; Pasquale and Somà 2010). Their low costs and high sensitivity have made them a very attractive option for detecting a variety of physical and chemical quantities, including very small mass, gas concentration and temperature variations (Park et al. 2012; Lin and Wang 2006; Madinei et al. 2015). Measuring very small biomedical masses such as viruses, bacteria, biomolecules, DNA, or protein has always been a very challenging issue (Jafari et al. 2017; Baguet et al. 2019; Younis 2011). Various sensing mechanisms have been proposed for small mass sensing purposes; these methods include frequency shift in the resonance zone due to the added mass (Park et al. 2012; Chauhan and Ansari 2021; Chellasivalingam et al. 2020; Joshi et al. 2019) symmetry-breaking of the vibration (Baguet et al. 2019; Chellasivalingam et al. 2020) and nonlinear bifurcations (Meesala et al. 2020). From the sensing type point of view, various sensing mechanisms have been applied so far which include piezoelectric (Chellasivalingam et al. 2020; Joshi et al. 2019; Azizi et al. 2017; Kumar et al. 2011), piezoresistive (Chu et al. 2018), electrostatic (Baguet et al. 2019), magnetic (Jafari et al. 2017) and nanocrystalline ZnO thin films (Bhattacharyya et al. 2008). The dynamic range enhancement of MEMS mass sensors has also been studied. Considering the geometry of the model cantilever beams with and without a tip mass have been widely studied in the literature for various applications ranging from energy harvesting (Zhang et al. 2022; Ghavami et al. 2018) to mass detection (Kumar et al. 2011; Zhao et al. 2018). From the analysis of the motion equations point of view, various approaches including numerical integration, nonlinear perturbation techniques, isogeometric analysis (IGA), (Madinei et al. 2015; Phung-Van et al. 2017; Thanh et al. 2022; Cuong-Le et al. 2022). Zhao et al. (Zhao et al. 2018) proposed a piezoelectric-based mass sensor with enhanced sensitivity due to the operation in the nonlinear bi-stable regime. Yaqoob et al. (2022) proposed a MEMS mass sensor for analyte detection using multi-mode excitation of a resonator. Wasisto et al. (2022) proposed a phase-locked loop frequency tracking system for portable piezoresistive cantilever mass sensors; their proposed model offered a mass detection in the order of ng. Toledo et al. (2019) demonstrated the potential of a piezoelectric resonator to develop a low-cost sensor for detecting microscopic masses; their model was capable of measuring mass with a sensitivity of 8.8 Hz/ng. Setiono et al. (2020) proposed an electrothermally actuated piezoresistive mass sensor to measure and monitor the changes of mass concentration of carbon nano particles in air. Their experiments were performed on two kinds of piezoresistive cantilever sensors and tipless atomic force microscopy cantilevers where the quality factor of the electrothermally actuated sensor in the in-plane operational mode was considerably higher than the out-of-plane vibration mode. Pinto et al. (2019) represented the most important metrics in the characterization of the dynamic mass sensors; they also showed that the quality factor dominates the mass sensing sensitivity as it improves from the order of some pg in atmospheric pressure to approximately 100 fg in the vacuum condition. Nayfeh et al. (2010) developed a mathematical model for a resonant gas sensor with the structure of a cantilever beam with a tip-mass exposed to electrostatic actuation; they captured the periodic orbits in the steady state by the finite difference method and applied Melnikov analysis for the detection of the homoclinic point, but did not account for the inertial and geometric nonlinearities. Stachiv et al. (2022) developed a 3D finite element model to accurately predict the resonant frequency and the corresponding mode shapes of a nano cantilever beam and the bound analyte as the added mass; they studied the impact of the size, mass, and the position of the analyte mass on the resonant frequency and the vibrational modes of the model. Despite the great efforts and studies, especially in SARS and COVID-19 virus detection (Broughton et al. 2020; Chan et al. 2020; Chan et al. 2019), ultraprecise bio-detection is still a challenging problem in biomedical and engineering sciences. Wang et al. (2022) proposed a molecular electromechanical system (MolEMS) consisting of an aptamer probe bound to a flexible single-stranded DNA cantilever which was connected to a self-assembled stiff tetrahedral double-stranded DNS structure which enabled super sensitive detection of proteins and small molecules in biofluids. Biomass sensing, especially for disease diagnosis, is very demanding and a very challenging problem (Ihling et al. 2020) in the engineering field, especially when the measurement range is less than *pg* range.

As mentioned, biomass sensing in the bio-medical research field is a highly demanding topic; in this paper, we propose a super sensitive piezoresistive MEMS mass sensor/switch whose sensitivity is enhanced by taking advantage of nonlinearity and setting the orientation of the structure and the piezoresistive layer with respect to the crystallographic coordinates of the silicon such that the highest possible sensitivity is achieved. The model consists of a cantilever beam with a tip mass at the end. The system is excited in the vicinity of the catastrophic bifurcation point, once the added mass is deposited on the tip mass, due to frequency shift as a result of mass addition, the system exhibits a jump in the time response which is applied as a measure of the added mass. The nonlinear equation of the motion, which accounts for the effect of geometric and inertial nonlinearities and the nonlinear electrostatic excitation is discretized and the frequency response curves for the reduced order model are calculated using the shooting method.

## Modelling

**Fig. 1:**
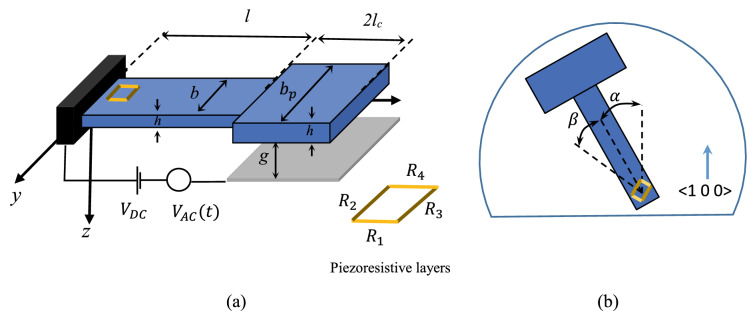
**a** Schematic of the piezoresistive mass sensor, **b** orientation of the device with respect to the crystallographic coordinate <1 0 0>

As shown in Fig. 1, the proposed mass sensor is composed of a silicon cantilever beam of length *l,* width *b* and thickness *h* with a tip mass of length *2l*_*c*_ and width *b*_*p*_. The coordinate system *x–y–z* is attached to the center of mass at the clamped end. The tip mass is excited by a DC voltage, *V*_*DC*_, superimposed by an AC voltage, *V*_*AC*_*.* The initial distance between the tip mass and the substrate is denoted by *g*. In order to compensate for the effect of the temperature change and counteract its effect on the resistance change of the piezoresistive layers, the piezoresistive layers are connected in a Wheatstone bridge configuration (Bao 2000; Fras et al. 2018; Zhao et al. 2016), as shown in Fig. 1(a). The piezoresistive layers are represented by ${R}_{1}, {R}_{2}, {R}_{3}, {R}_{4}$ where ${R}_{1}-{R}_{4}$ and ${R}_{2}-{R}_{3}$ are parallel to each other and formed by diffusion or ion-implantation on the surface of the cantilever beam. The orientation of the cantilever beam with respect to the crystallographic direction <1 0 0> is denoted by $\alpha$ and the angle of ${R}_{1}-{R}_{4}$ with respect to the direction of beam is denoted by $\beta$ (Fig. 1b).

The equation of the motion is given as (Azizi et al. 2022; Ali and Nayfeh 2004):

1 $$ \begin{aligned} & \rho A\frac{{\partial^{2} w}}{{\partial t^{2} }} + EI\frac{\partial }{\partial x}\left[ {\frac{{\partial^{3} w}}{{\partial x^{3} }} + \frac{\partial w}{{\partial x}}\left( {\frac{{\partial^{2} w}}{{\partial x^{2} }}} \right)^{2} + \frac{{\partial^{3} w}}{{\partial x^{3} }}\left( {\frac{\partial w}{{\partial x}}} \right)^{2} } \right] - J\frac{{\partial^{4} w}}{{\partial x^{2} \partial t^{2} }} \\ & \quad + \frac{1}{2}\frac{\partial }{\partial x}\left\{ {\frac{\partial w}{{\partial x}}\mathop \int \limits_{l}^{x} \rho A\frac{{\partial^{2} }}{{\partial t^{2} }}\left[ {\mathop \int \limits_{0}^{x} \left( {\frac{\partial w}{{\partial x}}} \right)^{2} dx} \right]dx} \right\} + c\frac{\partial w}{{\partial t}} = 0 \\ \end{aligned} $$

where *w* is the transverse displacement, *ρ* is mass density, *A* is cross-sectional area and *E* is modulus of elasticity. Here *I* is area moment of inertia of the cantilever beam with respect to neutral axis, *J* is the rotary inertia of the tip mass and *t* is time.

The boundary conditions are defined as (Nayfeh et al. 2010):

2 $$ \begin{aligned} w\left( {0,t} \right) & = 0,\quad \frac{\partial w}{{\partial x}}\left( {0,t} \right) = 0 \\ EI\frac{{\partial^{2} w}}{{\partial x^{2} }}\left( {l,t} \right) & = - \left( {M + \delta m} \right)l_{c} \frac{{\partial^{2} w}}{{\partial t^{2} }}\left( {l,t} \right) - \left( {\left( {M + \delta m} \right) l\_c + J_{M} } \right)\frac{{\partial^{3} w}}{{\partial x\partial t^{2} }}\left( {l,t} \right) + \frac{{\varepsilon b_{p} \left( {V_{DC} + V_{AC} } \right)^{2} }}{{2\left( {\frac{\partial w}{{\partial x}}\left( {l,t} \right)} \right)^{2} }} \\ & \quad \times \left[ {\frac{{2l_{c} \frac{\partial w}{{\partial x}}\left( {l,t} \right)}}{{g - w\left( {l,t} \right) - 2l_{c} \frac{\partial w}{{\partial x}}\left( {l,t} \right)}} - \ln \left( {\frac{{g - w\left( {l,t} \right)}}{{g - w\left( {l,t} \right) - 2l_{c} \frac{\partial w}{{\partial x}}\left( {l,t} \right)}}} \right)} \right], \\ EI\frac{{\partial^{3} w}}{{\partial x^{3} }}\left( {l,t} \right) & = \left( {M + \delta m} \right)\frac{{\partial^{2} w}}{{\partial t^{2} }}\left( {l,t} \right) + \left( {M + \delta m} \right)l_{c} \frac{{\partial^{3} w}}{{\partial x\partial t^{2} }}\left( {l,t} \right) - \frac{{\varepsilon b_{p} \left( {V_{DC} + V_{AC} } \right)^{2} }}{{2\frac{\partial w}{{\partial x}}\left( {l,t} \right)}} \\ & \quad \times \left[ {\frac{1}{{d - w\left( {l,t} \right) - 2l_{c} \frac{\partial w}{{\partial x}}\left( {l,t} \right)}} - \frac{1}{{d - w\left( {l,t} \right)}}} \right] \\ \end{aligned} $$

where *M* is the tip mass modelled as a rigid body with mass moment of inertia ${J}_{M}=\frac{1}{3}(M+\delta m){l}_{c}^{2}$, and $\delta m$ is the added mass which is assumed to be uniformly distributed on the tip mass, $\varepsilon$ is the permittivity of air. Introducing the following non-dimensional parameters (Azizi et al. 2016; Zamanzadeh et al. 2020),

3 $$ \begin{aligned} \hat{x} & = \frac{x}{l},\quad \hat{w} = \frac{w}{g},\quad \hat{t} = \frac{t}{T} ,\quad T = \sqrt {\frac{{\rho AL^{4} }}{EI}} , \\ \hat{c} & = \frac{{cl^{4} }}{EIT} ,\quad \hat{M} = \frac{{\left( {M + \delta m} \right)}}{\rho Al},\quad \hat{l}_{c} = \frac{{l_{c} }}{l} \\ \end{aligned} $$

and removing the hat notation, the dimensionless governing equation reduces to Azizi et al. (2022):

4 $$ \begin{aligned} & \frac{{\partial^{2} w}}{{\partial t^{2} }} + \frac{\partial }{\partial x}\left[ {\frac{{\partial^{3} w}}{{\partial x^{3} }} + \alpha_{1} \left( {\frac{\partial w}{{\partial x}}\left( {\frac{{\partial^{2} w}}{{\partial x^{2} }}} \right)^{2} + \frac{{\partial^{3} w}}{{\partial x^{3} }}\left( {\frac{\partial w}{{\partial x}}} \right)^{2} } \right)} \right] \\ & \quad + \frac{{\alpha_{1} }}{2}\frac{\partial }{\partial x}\left\{ {\frac{\partial w}{{\partial x}}\mathop \int \limits_{1}^{x} \frac{{\partial^{2} }}{{\partial t^{2} }}\left[ {\mathop \int \limits_{0}^{x} \left( {\frac{\partial w}{{\partial x}}} \right)^{2} dx} \right]dx} \right\} - \alpha_{2} \frac{{\partial^{4} w}}{{\partial x^{2} \partial t^{2} }} + c\frac{\partial w}{{\partial t}} = 0 \\ \end{aligned} $$

In Eq. (4), ${\mathrm{\alpha }}_{1}$ and ${\mathrm{\alpha }}_{2}$ are defined as:

5 $$ {\upalpha }_{1} = \frac{{g^{2} }}{{l^{2} }},\quad {\upalpha }_{2} = \frac{{J_{M} }}{{\rho A l^{2} }} $$

The associated boundary conditions in non-dimensional form reduce to Nayfeh et al. (2010):

6 $$ \begin{aligned} w\left( {0,t} \right) & = 0,\quad \frac{\partial w}{{\partial x}}\left( {0,t} \right) = 0, \\ \frac{{\partial^{2} w}}{{\partial x^{2} }}\left( {1,t} \right) & = - l_{c} M\frac{{\partial^{2} w}}{{\partial t^{2} }}\left( {1,t} \right) - \frac{4}{3}l_{c}^{2} M\frac{{\partial^{3} w}}{{\partial x\partial t^{2} }}\left( {1,t} \right) + \frac{{\alpha_{3} \left( {V_{DC} + V_{AC} } \right)^{2} }}{{\left( {\frac{\partial w}{{\partial x}}} \right)^{2} \left( {l,t} \right)}} \\ & \quad \times \left[ {\frac{{2l_{c} \frac{\partial w}{{\partial x}}\left( {1,t} \right)}}{{1 - w\left( {1,t} \right) - 2l_{c} \frac{\partial w}{{\partial x}}\left( {l,t} \right)}} - \ln \left( {\frac{{1 - w\left( {1,t} \right)}}{{1 - w\left( {1,t} \right) - 2l_{c} \frac{\partial w}{{\partial x}}\left( {1,t} \right)}}} \right)} \right] , \\ \frac{{\partial^{3} w}}{{\partial x^{3} }}\left( {1,t} \right) & = M\frac{{\partial^{2} w}}{{\partial t^{2} }}\left( {1,t} \right) + Ml_{c} \frac{{\partial^{3} w}}{{\partial x\partial t^{2} }}\left( {1,t} \right) - \frac{{\alpha_{3} \left( {V_{DC} + V_{AC} } \right)^{2} }}{{\frac{\partial w}{{\partial x}}\left( {1,t} \right)}} \times \left[ {\frac{1}{{1 - w\left( {1,t} \right) - 2l_{c} \frac{\partial w}{{\partial x}}\left( {1,t} \right)}} - \frac{1}{{1 - w\left( {1,t} \right)}}} \right] \\ \end{aligned} $$

where $${\alpha }_{3}=\frac{\varepsilon {b}_{p}{l}^{4}}{2EI{d}^{3}}$$.

Equation (4) is a homogenous partial differential equation (PDE) subjected to non-homogenous boundary conditions. To solve this equation, one possibility is to discretize the equation and then numerically integrate it over time with updated shape functions in each time step which requires a huge amount of computational time. The other possibility is to apply the Galerkin discretization method based on the linear mode shapes to the extended Hamiltonian (Nayfeh et al. 2010; Phung-Van et al. 2019) which yields a nonlinear non-homogenous ordinary differential equation (ODE). The other method is to apply the Galerkin discretization to the Lagrangian (Firoozy et al. 2017) which results in a number of non-homogenous ODEs. The latter approach is adopted in this study to derive the governing reduced order model. The shape functions are assumed to be linear which satisfy the PDE with only the linear terms retained (Eq. 4) subjected to non-homogenous linear boundary conditions (Eq. 6). For this purpose, the kinetic and the potential energies are given as:

7 $$ \begin{aligned} K & = \frac{1}{2}\left( {\rho A} \right)\mathop \int \limits_{0}^{1} \left[ {\frac{1}{4}\frac{{g^{4} }}{{lT^{2} }}\left( {\frac{\partial }{\partial t}\mathop \int \limits_{0}^{s} \left( {\frac{\partial w}{{\partial s}}} \right)^{2} ds} \right)^{2} + \frac{{g^{2} l}}{{T^{2} }}\left( {\frac{\partial w}{{\partial t}}} \right)^{2} } \right]ds \\ & \quad + \frac{1}{2}M_{t} \left[ {\left( {\frac{1}{2}\frac{{g^{2} }}{lT}\frac{\partial }{\partial t}\mathop \int \limits_{0}^{1} \left( {\frac{\partial w}{{\partial s}}} \right)^{2} ds + l_{c} \frac{{g^{2} }}{{l^{2} T}}\frac{\partial w}{{\partial s}}\frac{{\partial^{2} w}}{\partial s\partial t}\left( {1,t} \right)} \right)^{2} + \left( {\frac{g}{T}\frac{\partial w}{{\partial t}} + l_{c} \frac{g}{Tl}\frac{{\partial^{2} w}}{\partial t\partial s}\left( {1,t} \right)} \right)^{2} } \right] \\ & \quad + \frac{1}{2}J_{M} \left[ {\frac{{g^{2} }}{{T^{2} l^{2} }}\left( {\frac{{\partial^{2} w}}{\partial t\partial s}\left( {1,t} \right)} \right)^{2} \left( {1 + \left( {\frac{g}{l}\frac{\partial w}{{\partial s}}\left( {1,t} \right)} \right)^{2} } \right)} \right] \\ U & = \frac{1}{2}EI\frac{{g^{2} }}{{l^{3} }}\mathop \int \limits_{0}^{1} \left( {\frac{{\partial^{2} w}}{{\partial s^{2} }}} \right)^{2} \left( {1 + \frac{{g^{2} }}{{l^{2} }}\left( {\frac{\partial w}{{\partial s}}} \right)^{2} } \right)ds \\ & \quad - \frac{1}{2}\varepsilon b_{p} \left( {V_{DC} + V_{AC} } \right)^{2} \mathop \int \limits_{0}^{1} \frac{{2l_{c} d\tau }}{{g - gw\left( {1,t} \right) - \frac{g}{l}2l_{c} \frac{\partial w}{{\partial s}}\left( {1,t} \right)\tau }} \\ \end{aligned} $$

Substituting the expression:

8 $$ w\left( {x,t} \right) = \mathop \sum \limits_{i = 1}^{n} \varphi_{i} \left( x \right)q_{i} \left( t \right) $$

into the Lagrangian ($L=K-U$), applying the Galerkin method, and then deriving the motion equations using $\frac{d}{dt}\left(\frac{\partial L}{\partial \dot{q}}\right)-\frac{\partial L}{\partial q}+\frac{\partial D}{\partial \dot{q}}=0$ yields the following nonlinear ODE, for a single term $(\mathrm{i}.\mathrm{e}. n=1)$ subjected to the initial conditions. Hence (dropping the subscripts on *q* and$\varphi$)

9 $$ \left( {1 + \frac{\alpha }{\beta }q^{2} } \right)\ddot{q} + \left( {\frac{\gamma }{\beta }} \right)q + \left( {\frac{2\theta }{\beta }} \right)q^{3} - \frac{\alpha }{\beta }q\dot{q}^{2} = - \frac{\partial \Gamma }{{\partial q}}\frac{1}{2\beta } $$

where:

10 $$  \begin{aligned}   \alpha  &  = \frac{1}{2}\rho A\frac{{g^{4} }}{{lT^{2} }}\int\limits_{0}^{1} {\left( {\int\limits_{0}^{s} {\varphi ^{{\prime 2}} ds} } \right)^{2} ds}  + \frac{1}{2}J_{M} \frac{{g^{4} }}{{l^{4} T^{2} }}\varphi ^{{\prime 2}} \left( 1 \right) \\     &  + \frac{1}{2}M_{t} \left( \begin{gathered}   \frac{{g^{4} }}{{l^{2} T^{2} }}\left( {\int\limits_{0}^{1} {\varphi ^{{\prime 2}} ds} } \right)^{2}  + l_{c}^{2} \frac{{g^{4} }}{{l^{4} T^{2} }}\varphi ^{{\prime 4}} \left( 1 \right) \hfill \\    + 2l_{c} \frac{{g^{4} }}{{l^{3} T^{2} }}\varphi ^{{\prime 2}} \left( 1 \right)\int\limits_{0}^{1} {\varphi ^{{\prime 2}} ds}  \hfill \\  \end{gathered}  \right) \\    \beta  &  = \frac{1}{2}\rho A\frac{{g^{2} }}{{T^{2} }}l\int\limits_{0}^{1} {\varphi ^{2} ds}  \\     &  + \frac{1}{2}M_{t} \left( {\frac{{g^{2} }}{{T^{2} }}\varphi ^{2} \left( 1 \right) + l_{c}^{2} \frac{{g^{2} }}{{l^{2} T^{2} }}\varphi ^{{\prime 2}} \left( 1 \right) + 2\frac{{g^{2} }}{{lT^{2} }}l_{c} \varphi ^{\prime } \left( 1 \right)\varphi \left( 1 \right)} \right) \\     &  + \frac{1}{2}J_{M} \left( {\frac{{g^{2} }}{{l^{2} T^{2} }}\varphi ^{{\prime 2}} \left( 1 \right)} \right) \\    \gamma  &  = \frac{1}{2}EI\frac{{g^{2} }}{{l^{3} }}\int\limits_{0}^{1} {\varphi ^{{\prime \prime 2}} ds}  \\    \theta  &  = \frac{1}{2}EI\frac{{g^{4} }}{{l^{5} }}\int\limits_{0}^{1} {\varphi ^{{\prime \prime 2}} \varphi ^{{\prime 2}} ds}  \\    \Gamma  &  =  - \frac{{\varepsilon b_{p} \left( {V_{{DC}}  + V_{{AC}} } \right)^{2} l_{c} }}{g}\int\limits_{0}^{1} {\frac{{d\upsilon }}{{\left( {1 - q\varphi \left( 1 \right) - \frac{{2l_{c} }}{l}q\varphi ^{\prime } \left( 1 \right)\upsilon } \right)}}}  \\  \end{aligned}   $$

In Eq. (10), $\upsilon$ is a dummy parameter to carry out the integral over the length of the tip mass. For the numerical solution, the phase space variables are defined (Madinei et al. 2015; Meng et al. 2022; Wang et al. 2022) and the governing nonlinear ODEs are integrated over the time to get the time response.

## Resistivity tensor and orientation definition

Despite the isotropic and orientation-independent mechanical properties of silicon, the piezoresistive coefficient for some single crystalline semiconductors such as silicon and germanium is dependent on the orientation and accordingly anisotropic (Chan et al. 2020). The stress tensor ($\widetilde{{\varvec{T}}}$) in crystalline material causes a change in the resistivity tensor ($\rho ).$ The relation between the resistivity tensor and the stress tensor is given as (Bao 2000):

11 $$\left(\begin{array}{c}{\rho }_{1}\\ {\rho }_{2}\\ {\rho }_{3}\\ {\rho }_{4}\\ {\rho }_{5}\\ {\rho }_{6}\end{array}\right)=\left(\begin{array}{c}{\rho }_{0}\\ {\rho }_{0}\\ {\rho }_{0}\\ 0\\ 0\\ 0\end{array}\right)+{\rho }_{0}\left[\begin{array}{cccccc}{{\varvec{\pi}}}_{11}& {{\varvec{\pi}}}_{12}& {{\varvec{\pi}}}_{13}& {{\varvec{\pi}}}_{14}& {{\varvec{\pi}}}_{15}& {{\varvec{\pi}}}_{16}\\ {{\varvec{\pi}}}_{21}& {{\varvec{\pi}}}_{22}& {{\varvec{\pi}}}_{23}& {{\varvec{\pi}}}_{24}& {{\varvec{\pi}}}_{25}& {{\varvec{\pi}}}_{26}\\ {{\varvec{\pi}}}_{31}& {{\varvec{\pi}}}_{32}& {{\varvec{\pi}}}_{33}& {{\varvec{\pi}}}_{34}& {{\varvec{\pi}}}_{35}& {{\varvec{\pi}}}_{36}\\ {{\varvec{\pi}}}_{41}& {{\varvec{\pi}}}_{42}& {{\varvec{\pi}}}_{43}& {{\varvec{\pi}}}_{44}& {{\varvec{\pi}}}_{45}& {{\varvec{\pi}}}_{46}\\ {{\varvec{\pi}}}_{51}& {{\varvec{\pi}}}_{52}& {{\varvec{\pi}}}_{53}& {{\varvec{\pi}}}_{54}& {{\varvec{\pi}}}_{55}& {{\varvec{\pi}}}_{66}\\ {{\varvec{\pi}}}_{61}& {{\varvec{\pi}}}_{62}& {{\varvec{\pi}}}_{63}& {{\varvec{\pi}}}_{64}& {{\varvec{\pi}}}_{65}& {{\varvec{\pi}}}_{66}\end{array}\right]\left(\begin{array}{c}{T}_{1}\\ {T}_{2}\\ {T}_{3}\\ {T}_{4}\\ {T}_{5}\\ {T}_{6}\end{array}\right)$$

In Eq. (11) the ${T}_{i}, i=\mathrm{1,2}\dots 6$ and ${\rho }_{i}, i=\mathrm{1,2}\dots 6$ are the components of the second rank stress and resistivity tensors and are related by the piezoresistive coefficient tensor (${\varvec{\pi}}$) which is a tensor of fourth rank. ${T}_{i}$ and ${\rho }_{i}$ are represented in matrix notation as:

12 $$\widetilde{\mathbf{T}}=\left[\begin{array}{ccc}{\mathrm{T}}_{1}& {\mathrm{T}}_{6}& {\mathrm{T}}_{5}\\ {\mathrm{T}}_{6}& {\mathrm{T}}_{2}& {\mathrm{T}}_{4}\\ {\mathrm{T}}_{5}& {\mathrm{T}}_{4}& {\mathrm{T}}_{3}\end{array}\right]\uprho =\left[\begin{array}{ccc}{\uprho }_{1}& {\uprho }_{6}& {\uprho }_{5}\\ {\uprho }_{6}& {\uprho }_{2}& {\uprho }_{4}\\ {\uprho }_{5}& {\uprho }_{4}& {\uprho }_{3}\end{array}\right]$$

In the crystallographic coordinate system for silicon there are only three non-zero independent components for the piezoresistive coefficients which are ${{\varvec{\pi}}}_{11}={{\varvec{\pi}}}_{22}={{\varvec{\pi}}}_{33},\boldsymbol{ }{\boldsymbol{ }{\varvec{\pi}}}_{12}={{\varvec{\pi}}}_{21}={{\varvec{\pi}}}_{13}={{\varvec{\pi}}}_{31}={{\varvec{\pi}}}_{23}={{\varvec{\pi}}}_{32}$ and ${{\varvec{\pi}}}_{44}={{\varvec{\pi}}}_{55}={{\varvec{\pi}}}_{66}$. The components of the stress and resistivity tensor in another orientation rather than the crystallographic coordinate system are determined based on the coordinate transformation of the second order tensors; however the piezoresistive coefficient tensor obeys the corresponding rule for fourth-order tensors. The components of the piezoresistive tensor for both p-Si and n-Si are given in Table 1 (Smith 1954).

**Table 1:** Components of the piezoresistive tensor of silicon (in ${10}^{-11}/Pa$)

	${\pi }_{11}$	${\pi }_{12}$	${\pi }_{44}$
p-Si	6.6	− 1.1	138.1
n-Si	− 102.2	53.4	− 13.6

As the piezoresistive layers are positioned on the surface of the structure, in most applications the plane stress condition (${T}_{3}={T}_{4}={T}_{4}={T}_{5}=0$) holds and accordingly the component of the resistivity tensor along the length of the piezoresistive layers (two terminals are located at either end of the terminals) reduces to:

13 $$ \rho_{1} = \rho_{0} \left( {1 + {\varvec{\pi}}_{11} T_{1} + {\varvec{\pi}}_{12} T_{2} + {\varvec{\pi}}_{16} T_{6} } \right) $$

Equation (13) implies that the sensitivity along direction 1, is${\left(\Delta \rho /{\rho }_{0}\right)}_{1}={{\varvec{\pi}}}_{11}{T}_{1}+{{\varvec{\pi}}}_{12}{T}_{2}+{{\varvec{\pi}}}_{16}{T}_{6}$. Here ${T}_{1}$, ${T}_{2}$ and ${T}_{6}$ are longitudinal, transverse and shear stresses in a two dimensional element in which the longitudinal direction lies along the length of the piezoresistive layer; for simplicity the corresponding sensitivity is modified as ${\left(\Delta \rho /\rho \right)}_{l}={{\varvec{\pi}}}_{{\varvec{l}}}{T}_{l}+{{\varvec{\pi}}}_{{\varvec{t}}}{T}_{t}+{{\varvec{\pi}}}_{{\varvec{s}}}{T}_{s}$ where *l*, *s*, and *t* refer to the longitudinal, transverse and shear respectively; the components of the piezoresistive coefficients and stress tensors are computed based on determining the $\alpha$ and $\beta$ angles and the tensor transformation rules corresponding to the second and fourth order tensors respectively.

## Results and discussions

The geometric and mechanical properties of the studied model are given in Table 2.

**Table 2:** Mechanical and electrical properties of the studied model

$l$	250 μm
$b$	5 μm
$h$	1.5 μm
$g$	4 μm
${l}_{c}$	25 μm
${b}_{p}$	20 μm
$E$	169 GPa
$\rho$	2300 Kg/m^3^

**Fig. 2:**
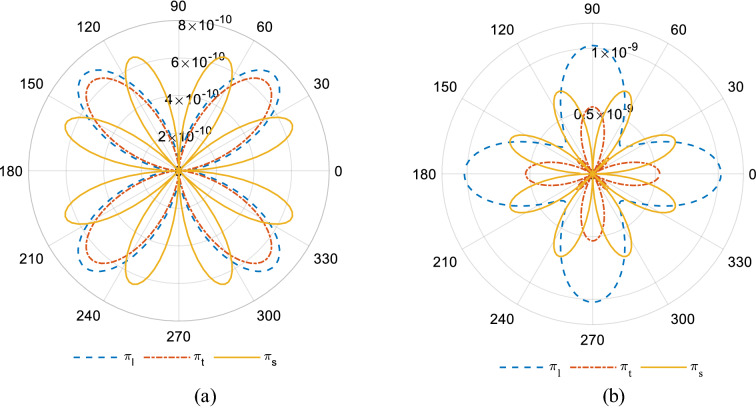
Dependency of the piezoresistive coefficients in terms of $\alpha$, **a** p-Si, **b** n-Si

Considering the coefficients of the piezoresistive tensor in the crystallographic coordinate system ($\alpha =0$), the orientation dependency of the piezoresistive coefficients in terms of $\alpha$ for both p-Si and n-Si are illustrated in Fig. 2.

**Fig. 3:**
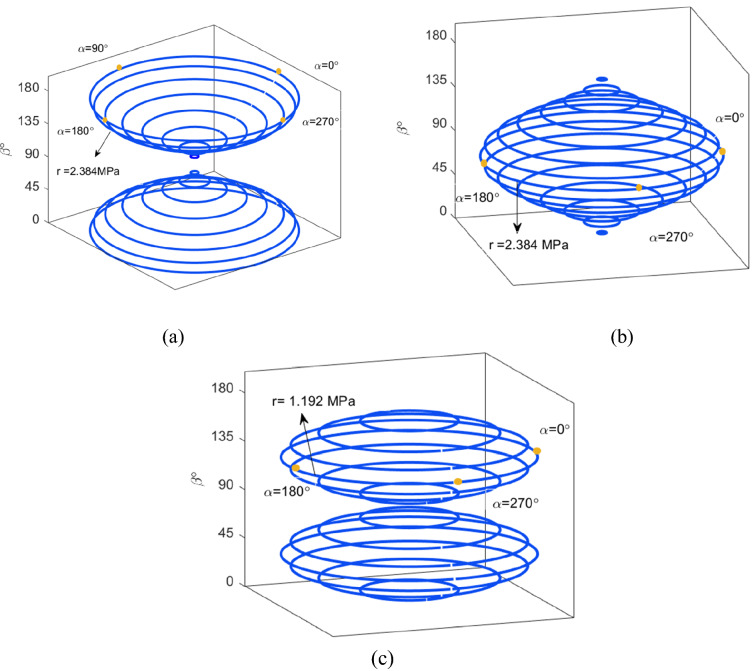
The components of stress tensor distribution (for each value of $\beta$ on the vertical axis $\alpha$ varies from ${0}^{^\circ }$ to${ 360}^{^\circ }$) **a** ${\sigma }_{l}$, **b**${\sigma }_{t}$, **c** ${\tau }_{s}$

The longitudinal, transversal and shear stress distribution for ${R}_{1}$ and ${R}_{4}$ in terms of $\alpha$ and $\beta$ are shown in cylindrical coordinates in Fig. 3; here, we assume for a horizontal cross-section (a fixed $\beta$), $\alpha$ varies in the range of ${0}^{^\circ }$ to ${360}^{^\circ }$. The components of stress tensor for the pair of ${R}_{2}$ and ${R}_{3}$ can be determined by adding ${90}^{^\circ }$ to the corresponding $\beta$ of the pair ${R}_{1}$ and ${R}_{4}$.

As illustrated, for $\beta =0$, the longitudinal stress ${\sigma }_{l}$ has a maximum which does not change with the variation of $\alpha$. As $\beta$ increases, ${\sigma }_{l}$ reduces while ${\sigma }_{t}$ increases. For $\beta ={90}^{^\circ }$, ${\sigma }_{l}$ is zero while ${\sigma }_{t}$ becomes maximum. The variation of the shear stress in terms of $\alpha$ and $\beta$ is given in Fig. 3c which shows that the shear stress is maximum for $\beta ={45}^{^\circ }$.

**Fig. 4:**
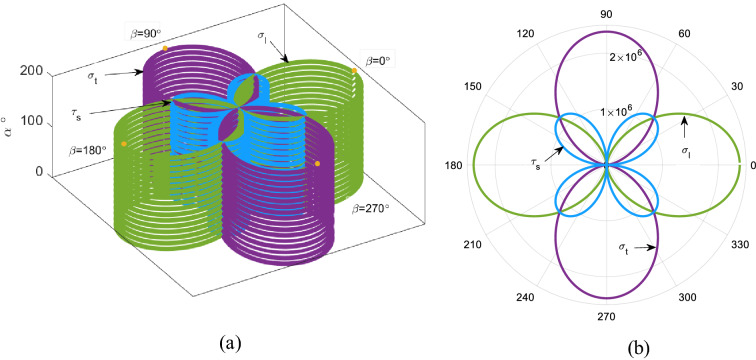
The components of stress tensor distribution (for each value of $\alpha$ on the vertical axis $\beta$ varies from ${0}^{^\circ }$ to ${360}^{^\circ }$) **a** three-dimensional view, **b** top view

Each horizontal cross section in Fig. 4 shows the variation of the stress tensor components for a fixed $\alpha$ while $\beta$ varies from ${0}^{^\circ }$ to ${360}^{^\circ }$. As illustrated, regardless of $\alpha$, the maximum value for ${\sigma }_{l}$ occurs at $\beta =0$, however the transverse and shear stresses reach their maximum values at $\beta ={90}^{^\circ }$ and $\beta ={45}^{^\circ }$, respectively.

**Fig. 5:**
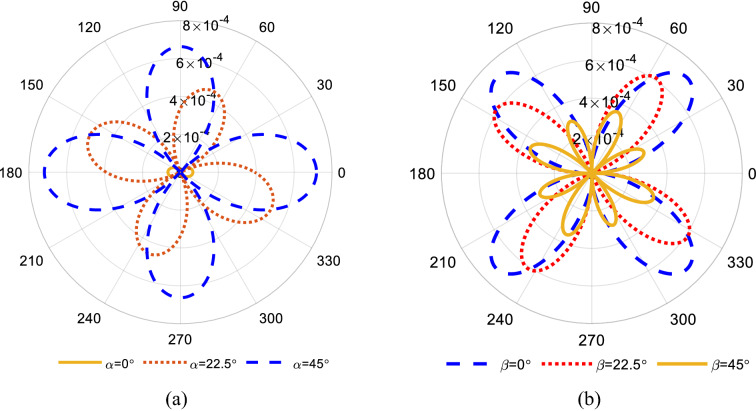
**a** Sensitivity assuming p-Si and 1 MPa uniaxial stress along the length of the cantilever beam, **a** fixed $\alpha$ and variable $\beta$, **b** fixed $\beta$ and variable $\alpha$

As discussed in the previous section, the sensitivity depends on stress and resistivity tensors. Considering a p-Si and assuming a 1 MPa uniaxial tensile stress along the length of the beam, we examine the sensitivity of the *R*_1_ and *R*_4_ resistors to determine the most sensitive orientation in terms of $\alpha$ and $\beta$. Figures 5a and b show the corresponding sensitivity for fixed $\alpha$ and variable $\beta$ and for fixed $\beta$ and variable $\alpha$, respectively. The maximum sensitivity corresponds to $\alpha ={45}^{^\circ }$ for an orientation of $<1 1 0>$ and $\beta ={0}^{^\circ }$ which are set as the desired orientations in the rest of this study.

**Fig. 6:**
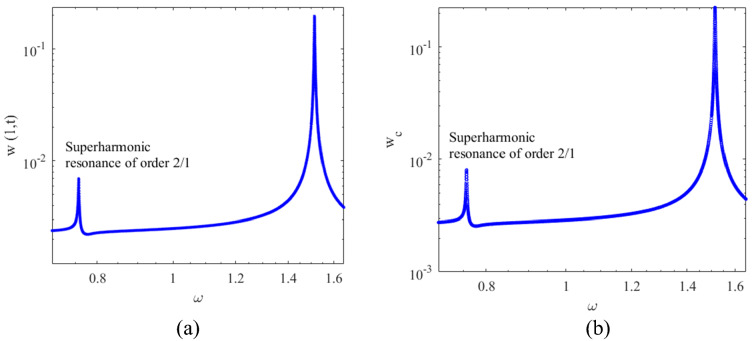
Frequency response corresponding to **a** $x=1$ on the cantilever beam and **b** the center of the tip mass

The frequency response curves of the cantilever beam in the vicinity of both superharmonic and primary resonances are shown in Fig. 6 for ${V}_{DC}=1v$ and ${V}_{AC}=0.1v$.

**Fig. 7:**
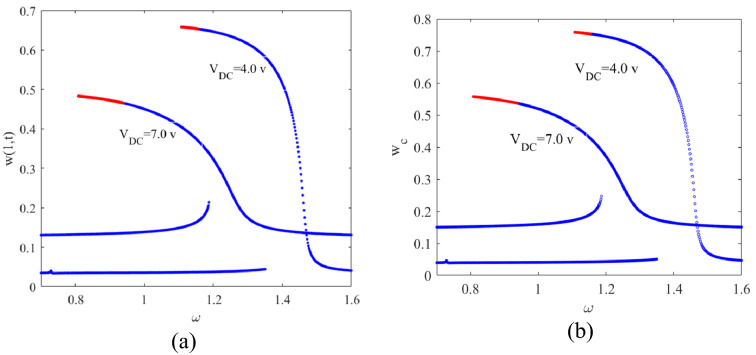
Frequency responses corresponding to **a** $x=1$ on the cantilever beam and **b** the center of the tip mass

Various sources of nonlinearity such as electrostatic, inertial, and geometric nonlinearity are present, however, electrostatic nonlinearity dominates the response. Electrostatic nonlinearity is in the form of quadratic nonlinearity and accordingly doubling and halving the excitation frequency mechanisms are active in the system; This ends up with super-harmonic and sub-harmonic nonlinear resonance zones on the frequency response curves of the system. Doubling of the excitation frequency, activates of the primary resonance of the system when the micro beam is excited by half of the frequency of the primary resonance. The existence of frequency doubling (in case of super-harmonic resonance) and frequency halving (in case of sub-harmonic resonance) in the dynamics of the system cause this nonlinear behaviour. This suggests that the amplitude of the motion should be increased to enhance the nonlinearity which accordingly produces nonlinear bifurcation points in the frequency domain; these bifurcation points are likely to exhibit super sensitivity for mass detection purposes. Figure 7 illustrates the frequency response curves for two different values of ${V}_{DC}$ and for ${V}_{AC}=0.1V$.

**Fig. 8:**
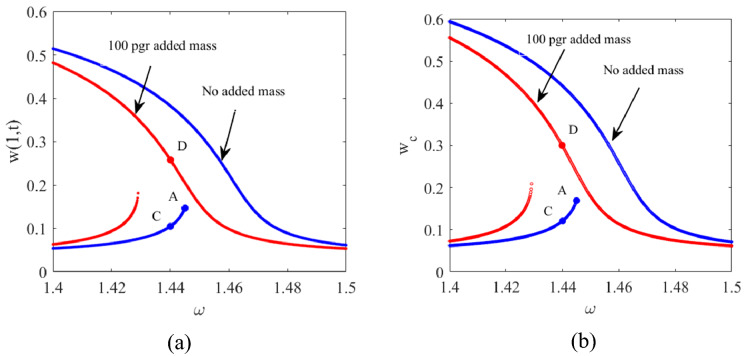
Frequency responses corresponding to **a** $x=1$ on the cantilever beam and **b** the center of the tip mass

By increasing ${V}_{DC}$, the bistable solutions approach each other and accordingly the higher amplitude branch of the solution for ${V}_{DC}=7\mathrm{ V}$ falls under the one corresponding to ${V}_{DC}=4\mathrm{ V}$. Once the excitation frequency is increased, the lower amplitude branch of the solution loses stability through a cyclic fold bifurcation point (Azizi et al. 2022). This point is where the stable and unstable manifolds intersect, and the system undergoes a sudden jump; the aim is to operate the system close to this bifurcation to take advantage of the sudden jump which offers a super sensitive regime for mass detection. Here we excite the system in the vicinity of the bifurcation point. The reason to not to operate exactly at the bifurcation point is because of the super sensitivity to very small disturbances since the low amplitude stable solution disappears beyond the bifurcation point. The consequence of even very small disturbances at point *A* in Fig. 8 might be a loss of stability and, accordingly a jump to a higher stable branch. The frequency response curves are shown in Fig. 8 for two different cases. The first case is in the absence of the added mass; whereas in the second case it is assumed that a 100 pg mass is uniformly deposited on the tip mass which shifts the frequency response curve toward the left and this means that any excitation frequency left of the bifurcation point which used to be on the lower stable manifold of the bistable solution, might remain right of the bifurcation point after the mass deposition and accordingly settle on the upper stable branch of the frequency response curve.

**Fig. 9:**
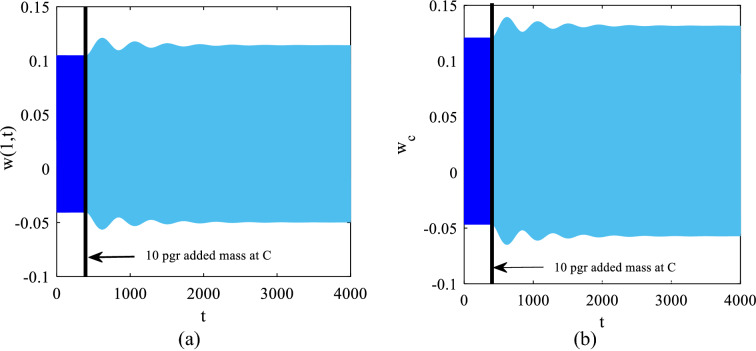
Time responses before and after 10 pg added mass **a** $x=1$ on the cantilever beam and **b** the center of the tip mass

In this section, we excite the tip mass with the frequency $\omega =1.44$ ($13.244 \mathrm{kHz}$) corresponding to point *C* which is on the left of bifurcation point *A* ($\omega =1.445$ ($13.290 \mathrm{kHz}$)) with the electrostatic excitation ${V}_{AC}=0.1\mathrm{ V}$, ${V}_{DC}=4.0\mathrm{ V}$. Figure 9 illustrates the time responses of both the tip of the cantilever beam and the center of the tip mass; here we assume that a uniformly added mass 10 pg is deposited on the tip mass at point *C*. The added mass shifts the frequency response curve to the left and accordingly, the excitation frequency approaches the bifurcation point on the modified frequency response curve. As represented here the added mass is not big enough to push the excitation frequency to the right of bifurcation point *A* and accordingly enable the jump to the higher amplitude stable solution. Once the 10 pg mass is deposited on the tip mass, the amplitude of the motion increases slightly which is attributed to the approach of the excitation frequency to the resonance zone but not to the jump.

**Fig. 10:**
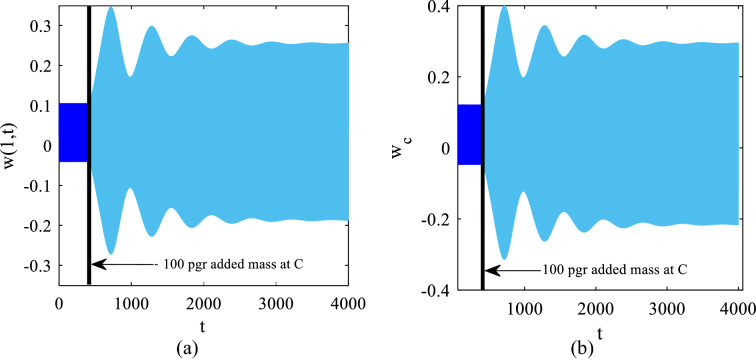
Time responses before and after 100 pg added mass **a** $x=1$ on the cantilever beam and **b** the center of the tip mass

Figure 10 gives the time responses of the cantilever tip and the center of the tip mass before and after the deposition of the 100 pg added mass.

**Fig. 11:**
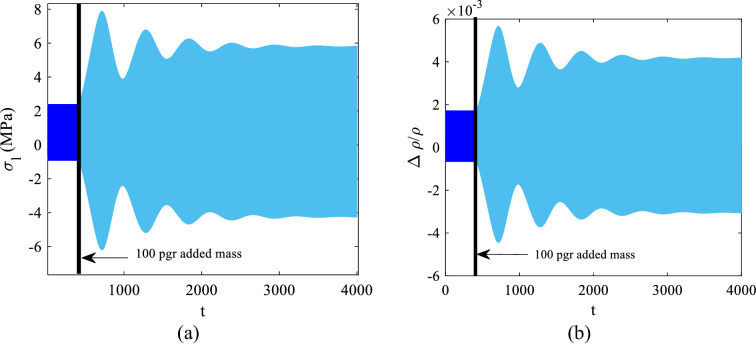
Time responses before and after deposition of 100 pg added mass, **a** ${\sigma }_{l}$ along the ${R}_{1}$ and ${R}_{4}$ resistances **b** Sensitivity for ${R}_{1}$ and ${R}_{4}$ resistances

With the deposition of the 100 pg added mass, the frequency response curve moves to the left more than the distance of the excitation frequency from the bifurcation point and accordingly, the system jumps through point *D* (Fig. 8) as the only possible stable response to which it eventually settles. The corresponding longitudinal normal stress ${\sigma }_{l}$ and sensitivity variations along the ${R}_{1}$ and ${R}_{4}$ resistances are illustrated in Fig. 11.

**Fig. 12:**
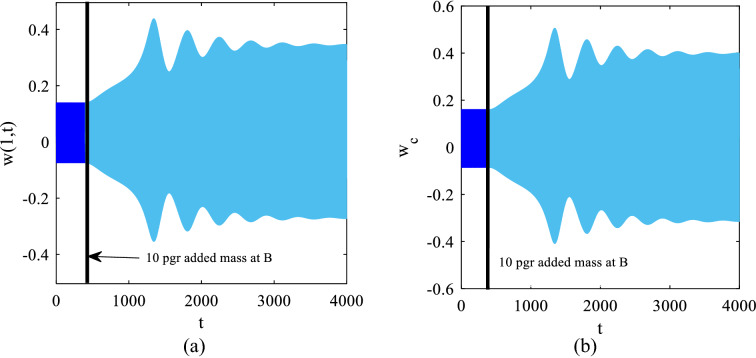
Time responses before and after 10 pg added mass **a** $x=1$ on the cantilever beam and **b** the center of the tip mass

We demonstrated that for the response to jump to the higher stable branch of the solution, the added mass needs to be more than the minimum required to trigger the bifurcation. It was shown that 10 pg added mass does not trigger the bifurcation in case the system is set to operate at *C* on the frequency domain; however, Fig. 12 shows that a jump is trigger when the system is set to initially operate at point *B* (shown in Fig. 8) with $\omega =1.4445$ ($13.285 \mathrm{kHz}$) which is 4.6 Hz from the bifurcation point *A*.

## Conclusion

In this paper, the nonlinear dynamics of a cantilever microbeam with a tip mass for mass sensing was investigated. The tip mass was subjected to electrostatic actuation as a combination of DC and AC voltages. Four piezoresistive layers in a Whetstone bridge configuration were connected to each other in the vicinity of the clamped end which undergoes the maximum normal stress. Since silicon is a non-isotropic material in terms of resistivity, we accounted for the effect of the orientation of the structure with respect to the crystallographic coordinates and its effect on the components of the resistivity tensor. The dependency of the sensitivity on the orientation of the piezoresistive layers with respect to the cantilever length was also investigated. We accounted for the geometric, inertial and electrostatic nonlinearities in the modelling. It was demonstrated that due to the nonlinearity of the response, there exists a cyclic fold bifurcation point on the frequency response curves where the stable and unstable branches of the solution on the frequency domain approach each other and they intersect at the cyclic fold bifurcation point beyond which both branches disappear and accordingly a jump occurs. It was shown that mass deposition moves the frequency response curves leftward in the frequency domain. Should the system be run to the left of the jump bifurcation, for an added mass higher than the threshold value, the operation frequency remains to the right of the bifurcation point, where system finds no stable low amplitude solutions and consequently jumps to the high amplitude stable response. We not only took the benefit of the nonlinearity to force the system to exhibit a sudden jump in the amplitude of the motion once the added mass is deposited on the tip mass, but also, we investigated the most sensitive direction of the piezoresistive layers with respect to the crystallographic orientation of the silicon. The study was performed for both p-Si and n-Si. The joint enhancement of the sensitivity due to the nonlinearity and orientation adjustment of the piezoresistive layers, offered a twofold sensitivity enhancement which is a promising improvement in the design of mass sensors for biological applications.

## Declarations

### Data availability

Data sharing not applicable to this article as no datasets were generated or analyzed during the current study.
